# The Minimum Legal Drinking Age: History, Effectiveness, and Ongoing Debate

**Published:** 1996

**Authors:** Traci L. Toomey, Carolyn Rosenfeld, Alexander C. Wagenaar

**Affiliations:** Traci L. Toomey, Ph.D., is a research associate with, Carolyn Rosenfeld, M.P.H., is a recent graduate of, and Alexander C. Wagenaar, Ph.D., is a professor in the Division of Epidemiology, University of Minnesota, Minneapolis, Minnesota

**Keywords:** minimum drinking age laws, evaluation, history of AOD public policy, public policy on AOD, adolescent, law enforcement, AOD availability, AOD sales, AODR (alcohol and other drug related) injury prevention, AODR mortality, traffic accident, drinking and driving

## Abstract

Minimum legal drinking age (MLDA) laws provide an example of how scientific research can support effective public policies. Between 1970 and 1975, 29 States lowered their MLDAs; subsequently, scientists found that traffic crashes increased significantly among teenagers. Alcohol use among youth is related to many problems, including traffic crashes, drownings, vandalism, assaults, homicides, suicides, teenage pregnancies, and sexually transmitted diseases. Research has demonstrated the effectiveness of a higher MLDA in preventing injuries and deaths among youth. Despite laws prohibiting the sale or provision of alcohol to people under age 21, minors can easily obtain alcohol from many sources. Increased MLDA enforcement levels and deterrents for adults who might sell or provide alcohol to minors can help prevent additional injuries and deaths among youth.

Science can play a critical role in developing effective policies to address health issues, including those focused on alcohol-related problems (Gordis 1991). In an ideal world, public policy development would be based on the identification of a problem and the scientific evidence of the factors that are most effective in reducing that problem. In the real world, however, public policy results from economic and political forces, which occasionally combine with good science. Minimum legal drinking age (MLDA) laws provide an example of how scientific research can support effective public policies. This article shows how science has influenced MLDA policies in the past and summarizes research contributing to the ongoing debate on the MLDA.

## History of the MLDA

Following the repeal of Prohibition, nearly all State laws restricting youth access to alcohol designated 21 as the minimum age for purchasing and consuming alcohol (Mosher 1980). Between 1970 and 1975, however, 29 States lowered the MLDA to age 18, 19, or 20. These changes occurred at the same time that minimum-age limits for other activities, such as voting, also were being lowered (Wechsler and Sands 1980). Scientists began studying the effects of the lowered MLDA, particularly focusing on traffic crashes, the leading cause of death among teenagers. Several studies in the 1970’s showed that traffic crashes increased significantly among teenagers after the MLDA was lowered (Cucchiaro et al. 1974; Douglass et al. 1974; Wagenaar 1983, 1993; Whitehead 1977; Whitehead et al. 1975; Williams et al. 1974).

With evidence that lower legal drinking ages were associated with more traffic crashes among youth, citizen advocacy groups led a movement to restore the MLDA to 21 in all States (Wolfson 1995). In response, 16 States increased their MLDA’s between September 1976 and January 1983 (Wagenaar 1983). Many States, however, resisted pressure from these groups and ignored Government incentives to raise their MLDA’s (King 1987). The Federal Government became concerned about the safety both of youth in States that had lower MLDA’s and of youth who lived in neighboring States. Persons who were below the MLDA in their own State could drive across State borders to purchase alcohol in a State with a lower MLDA and then return home, increasing the likelihood of being involved in traffic crashes.

Because the 21st amendment to the U.S. Constitution guaranteed States’ rights to regulate alcohol, the Federal Government could not mandate a uniform MLDA of 21. Instead, in 1984 the Federal Government passed the Uniform Drinking Age Act, which provided for a decrease in Federal highway funding to States that did not establish an MLDA of 21 by 1987 (King 1987). Faced with a loss of funding, the remaining States returned their MLDA’s to age 21 by 1988.

## Effectiveness of the MLDA

### Reductions in Drinking

Following the end of Prohibition, each State developed its own set of policies to regulate the distribution, sale, and consumption of alcohol. In addition to the MLDA, examples of other alcohol control policies include excise taxes, restrictions on hours and days of sales, and server training. Many of these other alcohol policies have only recently been evaluated (see Toomey et al. 1994 for a review of the research literature). Of all the alcohol control policies, MLDA policy has been the most studied. Since the 1970’s, at least 70 studies have explicitly examined the effects of either increases or decreases in the MLDA, with some studies using more robust research designs than others. MLDA policies may have been evaluated sooner and more often for a variety of reasons, including: (1) a growing concern about youth drinking and driving; (2) availability of archived, time-series data on traffic crashes; (3) the fact that many States first lowered, then raised, their MLDA’s; and (4) preliminary research showing the large effects of changes in MLDA’s. Thorough literature reviews by Wagenaar (1983, 1993), the United States General Accounting Office (1987), and Moskowitz (1989) provide detailed summaries of many of these studies. MLDA laws have been evaluated mostly in terms of how changing the MLDA affects rates of alcohol use and traffic crashes among youth.

Methods used to study the effect of the MLDA on alcohol use have varied widely, contributing to differences in conclusions among studies. For example, some studies used convenience samples, such as students in introductory psychology classes, whereas other studies used sophisticated, random sampling designs to obtain nationally representative samples. Wagenaar (1993) concluded that studies employing strong research and analytical designs typically observed increases in alcohol use among youth following a lowering of the MLDA. In contrast, when many States raised the MLDA, alcohol use among youth decreased.

Beer is the alcoholic beverage of choice for most youth. As a result, reduced rates of alcohol use among youth after the MLDA was increased were primarily evident in decreased rates of beer consumption (Berger and Snortum 1985). Rates of wine and distilled spirits use among youth did not change dramatically following the rise in the MLDA (Barsby and Marshall 1977; Smart 1977).

Opponents of the age-21 MLDA theorized that even if a higher MLDA reduced alcohol use among minors, drinking rates and alcohol-related problems would surge among those age 21 and older. In other words, opponents believed that a “rubber band” effect would occur: When youth turned 21, they would drink to “make up for lost time” and thus drink at higher rates than they would had they been allowed to drink alcohol at an earlier age. A study by O’Malley and Wagenaar (1991), however, refutes this theory. Using a national probability sample, O’Malley and Wagenaar found that the lower rates of alcohol use due to a high legal drinking age continued even after youth turned 21.

Although the MLDA’s effect on youth alcohol consumption is important, a key consideration is whether the MLDA ultimately affects the rates of alcohol-related problems. Alcohol use among youth is related to numerous problems, including traffic crashes, drownings, vandalism, assaults, homicides, suicides, teenage pregnancies, and sexually transmitted diseases. Alcohol use is reported in one-fifth to two-thirds of many of these problems (Howland and Hingson 1988; Plant 1990; Roizen 1982; Smith and Kraus 1988; Strunin and Hingson 1992). As drinking rates increase or decrease, rates of alcohol-related problems may change in response.

### Decreases in Traffic Crashes

Using various research methods, at least 50 studies have evaluated the effect of changes in the MLDA on traffic crashes (Wagenaar 1993). Some studies assessed policy changes in only one State, whereas others analyzed the MLDA’s effect across multiple States. These studies evaluated the effect of MLDA changes on a variety of outcomes, including total traffic crash fatalities for youth; drinking-driving convictions; crashes resulting in injuries; and single-vehicle nighttime crash fatalities (the crashes most likely to involve alcohol).

Most studies on the effect of lowering the MLDA found an increase in traffic crashes and traffic deaths among youth (Wagenaar 1993). Of the 29 studies completed since the early 1980’s that evaluated increases in the MLDA, 20 showed significant decreases in traffic crashes and crash fatalities. Only three clearly found no change in traffic crashes involving youth. The remaining six studies had equivocal results. Based on results from research studies such as these, the National Highway Traffic Safety Administration (NHTSA) estimated that in 1987 alone, 1,071 traffic crash fatalities were prevented because of the MLDA of 21 (NHTSA 1989).

Since 1984 researchers have been investigating whether changes in the MLDA also affect other alcohol-related problems. Of the four studies conducted to date that focused on other social and health consequences of alcohol use, three found an inverse relationship between the MLDA and alcohol-related problems: A higher legal drinking age was correlated with a lower number of alcohol problems among youth. The New York State Division of Alcoholism and Alcohol Abuse (1984) found a 16-percent decrease in rates of vandalism in four States that raised the MLDA. In a study of an increase of the MLDA in Massachusetts, Hingson and colleagues (1985) did not find significant changes in the rates of non-motor-vehicle trauma, suicide, or homicide. Smith (1986), however, found an increase in non-traffic-related hospital admissions following decreases in the MLDA in two Australian states. Jones and colleagues (1992) found lower rates of death caused by suicides, motor vehicle crashes, pedestrian accidents, and other injuries in States with higher MLDA’s. More research is needed to characterize the full effect of the MLDA on rates of alcohol-related injuries and on problems other than motor vehicle crashes.

## The Role of Enforcement

Research indicates that a higher MLDA results in fewer alcohol-related problems among youth and that the MLDA of 21 saves the lives of well over 1,000 youth each year (NHTSA 1989; Jones et al. 1992). What is compelling is that the effect of the higher MLDA is occurring with little or no enforcement. A common argument among opponents of a higher MLDA is that because many minors still drink and purchase alcohol, an MLDA of 21 does not work. The evidence shows, however, that although many youth still consume alcohol, they drink less and experience fewer alcohol-related injuries and deaths than they did under lower MLDA’s (Wagenaar 1993). A more appropriate discussion, therefore, is not whether the MLDA should again be lowered but whether the current MLDA can be made even more effective.

Despite laws prohibiting the sale or provision of alcohol to people under age 21, minors throughout the United States can easily obtain alcohol from many sources. Buyers who appear to be younger than 21 can successfully purchase alcohol from licensed establishments without showing age identification in 50 percent or more of their attempts (Forster et al. 1994, 1995; Preusser and Williams 1992). In addition, although many youth purchase alcohol themselves, most youth indicate that they generally obtain alcohol through social contacts over age 21 (Wagenaar et al. 1996*b*; Jones-Webb et al. in press). These social contacts—who include friends, siblings, parents, coworkers, and strangers approached outside of alcohol establishments—purchase alcohol and then either provide or sell it to minors.

Commercial establishments licensed to sell alcohol, as well as social sources, face potential criminal penalties, fines, license suspensions, and lawsuits for selling or providing alcohol to minors. So why do they still supply alcohol to youth? One reason is that policies are not actively enforced. For policies to deter specific behaviors effectively, people must believe that they have some chance of being caught and that they will face swift consequences for noncompliance (Gibbs 1975; Ross 1992). Wolfson and colleagues (1996*b*) found that only 38 percent of the alcohol merchants they surveyed thought it was likely that they would be cited for selling alcohol to a minor. Further research is needed to determine whether social sources are aware of their legal liability for providing alcohol to youth and whether they perceive a high likelihood of facing penalties for doing so.

Laws prohibiting the sale and provision of alcohol to minors are not well enforced (Wagenaar and Wolfson 1995), and systems for enforcing the legislation vary by State. Typically, however, enforcement systems use both State administrative agencies, usually called State Alcohol Beverage Control (ABC) agencies, and local law enforcement agencies, such as police departments and county sheriffs. Enforcement of MLDA laws has focused primarily on penalizing underage drinkers for illegal alcohol possession or consumption (Wagenaar and Wolfson 1995), an unintended and unanticipated consequence of the MLDA (Mosher 1995; Wolfson and Hourigan in press). For every 1,000 minors arrested for alcohol possession, only 130 establishments that sell alcohol to them have actions taken against them, and only 88 adults who purchase alcohol for minors face criminal penalties. Wagenaar and Wolfson (1994) estimate that only 5 of every 100,000 incidents of minors’ drinking result in a fine, license revocation, or license suspension of an alcohol establishment.

An in-depth review of enforcement actions in 295 counties in 4 States (Kentucky, Michigan, Montana, and Oregon) showed that in a 3-year period, 27 percent of the counties took no action against licensed establishments for selling alcohol to minors, and 41 percent of those counties made no arrests of adults who provided alcohol to minors (Wagenaar and Wolfson 1995). The States were selected for their diversity of alcohol-control systems and availability of data. Although the majority of counties took at least one action against alcohol establishments and adults who provided alcohol to youth, many did not take actions frequently.

As noted earlier, only a tiny proportion of incidents of minors’ drinking results in fines or other penalties for establishments that sell alcohol. Some reasons that enforcement agencies do not cite or arrest illegal providers include (1) perceived acceptance of underage drinking by community members, (2) lack of community encouragement to increase enforcement of the MLDA, and (3) lack of resources (Wolfson et al. 1995).

Given the low level of enforcement activity, it is not surprising that many adults do not hesitate to sell or give alcohol to minors. To create a deterrent effect, we need to increase the likelihood of facing negative consequences for illegally selling or providing alcohol to youth. One approach is to encourage ABC and local law enforcement agencies to increase enforcement against illegal alcohol providers. Preusser and colleagues (1994) found dramatic reductions in alcohol sales to minors (from 59 percent at baseline to 26 percent 1 year later) following an enforcement campaign involving three “sting operations” in which underage males attempted to purchase alcohol.

In addition to increasing enforcement of the MLDA, other procedures and policies can be implemented to improve the effectiveness of MLDA laws. To ensure that adults do not sell or provide alcohol to minors, both public and institutional policies can be developed that complement MLDA laws (Wagenaar et al. 1996*a*). Alcohol establishments, for example, can implement several policies and practices, including (1) requiring all alcohol servers to receive responsible service training on how to check age identification and refuse sales to teenagers, (2) establishing systems to monitor servers to prevent illegal sales to youth, and (3) posting warning signs (Wolfson et al. 1996*a*,*b*). Wolfson and colleagues (1996*a*,*b*) found that establishments adhering to these policies were less likely to sell alcohol to young women who appeared to be under age 21 and who did not present age identification.

## The Ongoing MLDA Debate

Despite an abundance of research demonstrating the effectiveness of the age-21 MLDA at saving lives and reducing alcohol-related problems, several States are again considering lowering their legal age limits for drinking. Louisiana’s MLDA of 21 was recently challenged in court on the premise that it violates the State’s constitutional law regarding age discrimination. Louisiana’s State Supreme Court concluded, however, that “. . . statutes establishing the minimum drinking age at a level higher than the age of majority are not arbitrary because they substantially further the appropriate governmental purpose of improving highway safety, and thus are constitutional” (Manuel v. State of Louisiana [La. 1996]). In other words, because the MLDA was based on empirical evidence that such laws saved lives, the court decided that the law was not arbitrary and thus did not violate Louisiana’s constitution. Despite the Louisiana decision, the MLDA of 21 also may be challenged in other States.

## Conclusion

The same arguments used to lower the MLDA 20 years ago are being used today (see sidebar, pp. 216–217). Despite ongoing debates about the MLDA, research demonstrates the effectiveness of a higher MLDA in preventing alcohol-related injuries and deaths among youth. As the MLDA’s were lowered, rates of injuries and deaths increased; when the MLDA’s were raised, injuries and deaths significantly decreased. The benefit of using environmental (i.e., external) approaches, such as the MLDA, is further supported by the fact that drinking rates were reduced even after youth turned age 21. In contrast, individual approaches (e.g., school-based programs) have generated only short-term reductions in underage drinking. This finding suggests that to create long-term changes in youth drinking and alcohol-related problems, strategies that change the environment should be used.

**The Public Debate Over the MLDA**

The public debate over reducing the legal drinking age has remained essentially unchanged since the minimum legal drinking age (MLDA) was first lowered in the 1970’s. Following are some frequently asked questions concerning the MLDA, along with answers based on the research findings to date.

**Question:** If States are the only entities that have the right to establish a minimum drinking age, does Federal legislation concerning this policy area infringe on State powers?

**Answer:** The initial movement to raise the MLDA to 21 was largely fueled by citizen action groups in several States, which raised their drinking ages before the Federal Government passed any legislation on the matter. Moreover, the Federal Government encouraged States to increase their MLDA’s to 21 to reduce traffic crashes caused by people driving to States that had lower MLDA’s. The Federal Government did not mandate the change. Polls continue to show strong public support for the drinking age of 21 (Wagenaar 1993*a*).

**Question:** Many Europeans let their children drink from an early age, and European countries do not have the same alcohol-related problems that we do. Therefore, how can people claim that MLDA’s are a major factor in helping to prevent alcohol problems in the United States?

**Answer:** Research confirms that European countries do experience alcohol-related problems. For example, European countries have rates of alcohol-induced diseases, such as cirrhosis of the liver, similar to (or higher than) the United States (Single 1984). Drunk driving among youth may not be as great a problem in Europe; compared with youth in the United States, European youth obtain their drivers’ licenses at an older age, are less able to afford automobiles, and more often use public transportation. Youth in Europe thus may be at lower risk of traffic crashes simply because they drive less frequently than their U.S. counterparts. Other alcohol-related problems are significant enough in Europe that those countries are examining the U.S. experience regarding MLDA policy and are initiating a debate over the most appropriate age for legal access to alcohol (Wagenaar 1993*a*).

In reviewing another country’s success with a given policy, one cannot simply compare international rates of alcohol-related problems without assessing the role of factors that contribute to the problems. Many cultural, political, and social conditions, which differ from country to country, affect drinking rates. The most robust research, although conducted in the United States, has shown a strong inverse relationship between MLDA and alcohol consumption and its related problems: As MLDA increases, alcohol-related problems among youth decrease. As MLDA changes occur in Europe, researchers will be able to determine more accurately the effect of a higher MLDA on alcohol-related problems among European youth.

**Question:** If a person is old enough to serve in the military, how can he or she not be old enough to buy alcohol?

**Answer:** Different activities have different ages of initiation: A person can drive at age 16, vote in elections and serve in the military at age 18, and serve as President at age 35. These restrictions are based on the requirements of the specific activities (e.g., motor skills, capacity for judgment, and experience) and take into account the risks and benefits of participation at different ages (Fell 1985). For example, research shows that at a given blood alcohol concentration, youth are more likely to be impaired than adults. Underage drinking is strongly related to serious public health problems, including injuries and death resulting from motor vehicle crashes, homicide, assault, and recreational injury. Consequently, policymakers and researchers have come to believe that risk both to youth and to society in general can be reduced by restricting people below age 21 from drinking.

**Question:** How can researchers be sure that the drop in rates of alcohol-related crashes among 19-and 20-year-olds following the increase in the MLDA to 21 was related to MLDA policy?

**Answer:** When the age-21 restriction was initiated, alcohol-involved highway crashes declined among 18- to 20-year-olds. This decline occurred with limited enforcement of the MLDA laws. The decline is therefore not attributable to drinking-driving enforcement and tougher penalties but directly results from lower consumption levels (O’Malley and Wagenaar 1991).

**Question:** If people cannot legally drink until they are 21, will they just drink more when they reach the MLDA?

**Answer:** Research indicates that the opposite is true (Wagenaar 1993*b*). In fact, early legal access to alcohol (i.e., at age 18) is associated with higher rates of drinking later in life. Research shows that when the MLDA is 21, people under age 21 drink less and continue to do so through their early twenties. Those who are inclined to drink do not “make up for lost time” after turning 21 (O’Malley and Wagenaar 1991).

—Carolyn Rosenfeld

ReferencesFellJCDrinking age 21: Facts, myths and fictionsAmerican Association for Automotive Medicine Quarterly Journal7121261985O’MalleyPMWagenaarACEffects of minimum drinking age laws on alcohol use, related behaviors and traffic crash involvement among American youth: 1976–1987Journal of Studies on Alcohol5254784911991194310510.15288/jsa.1991.52.478SingleEInternational perspectives on alcohol as a public health issueJournal of Public Health Policy623825919846470133WagenaarACMinimum drinking age lawsEncyclopedia of Drugs and Alcohol3151993aWagenaarACMinimum drinking age and alcohol availability to youth: Issues and research needsHiltonMEBlossGEconomics and the Prevention of Alcohol-Related ProblemsNational Institute on Alcohol Abuse and Alcoholism Research Monograph No. 25NIH Pub. No. 93–3513Bethesda, MDthe Institute1993b175200

Despite the MLDA of 21, minors still have easy access to alcohol from commercial and social sources. The observed benefits of the MLDA have occurred with little or no active enforcement; simply by increasing enforcement levels and deterring adults from selling or providing alcohol to minors, even more injuries and deaths related to alcohol use among youth can be prevented each year.
